# Achieving Diagnostic Excellence in Prenatal Diagnosis Through Patient-Reported Measures: Comment on "Achieving Diagnostic Excellence: Roadmaps to Develop and Use Patient-Reported Measures With an Equity Lens"

**DOI:** 10.34172/ijhpm.8921

**Published:** 2025-07-15

**Authors:** An Chen, Oona Tchitcherin, Kirsi Väyrynen, Paulus Torkki, Seppo Heinonen, Aydin Tekay

**Affiliations:** ^1^School of Public Health, Zhejiang Chinese Medical University, Zhejiang, China.; ^2^Department of Obstetrics and Gynecology, Helsinki University Hospital, Helsinki, Finland.; ^3^Department of Public Health, Faculty of Medicine, University of Helsinki, Helsinki, Finland.; ^4^Department of Obstetrics and Gynecology, Central Finland Central Hospital, Jyväskylä, Finland.

**Keywords:** Diagnostic Excellence, Patient-Reported Measures (PRMs), Prenatal Diagnosis, Diagnostic Uncertainty, Communication, Equity in Healthcare

## Abstract

McDonald and colleagues’ work "Achieving Diagnostic Excellence: Roadmaps to Develop and Use Patient-Reported Measures With an Equity Lens" emphasizes the critical role of patient-reported measures (PRMs) in achieving diagnostic excellence, with a focus on equity. PRMs capture patients’ experiences, symptoms, and concerns throughout the diagnostic process, enhancing accuracy and addressing uncertainties. In contexts like maternity care, PRMs have been applied to reveal and reduce communication gaps and alleviate anxiety, offering crucial support for improving diagnostic experiences and outcomes. This commentary explores the application of McDonald and colleagues’ visions and roadmaps to prenatal diagnosis, specifically focusing "prenatal screening and testing" (PreST), a complex process where diverse patient populations face challenges in understanding and responding to sequential test results. Tailored diagnosis-related PRMs can provide healthcare providers with tools to enhance shared decision-making, equitable communication, and patient satisfaction, leading to more inclusive and personalized diagnostic pathways.

## Patient-Reported Measures: Advancing Diagnostic Excellence by Addressing Variability in Patients

 The publication by McDonald et al^1^ “Achieving Diagnostic Excellence: Roadmaps to Develop and Use Patient-Reported Measures With an Equity Lens” offers a fresh perspective into the role of patient-reported measures (PRMs) in achieving diagnostic excellence, particularly through the lens of equity. It crafts a set of sample roadmaps of developing PRMs and incorporating them into diagnostic pathways with a focus on equity. McDonald and colleagues’ approach^1^ to diagnostic excellence is relevant in today’s healthcare environment, where disparities in diagnosis and care often lead to suboptimal outcomes, especially for marginalized populations. The focus on equity adds a vital layer to understanding diagnostic processes, making the research timely and essential.

 It has been widely acknowledged that PRMs, which capture patients’ perceptions, experiences, and concerns, serve as a crucial part of diagnostic evidence, significantly contributing to diagnostic excellence by identifying patient-specific nuances, providing a more comprehensive symptom profile, improving diagnostic accuracy, and addressing diagnostic uncertainty.^2^ However, a critical takeaway from McDonald and colleagues’ paper is the need to develop PRMs that capture the full scope of diagnostic outcomes and experiences, especially in populations prone to inequities.^1^ McDonald et al emphasizes that PRMs, when developed with an equity lens, can not only improve diagnostic outcomes but also help in recognizing and addressing systemic biases in the diagnostic process.^1^ Particularly, it highlights how PRMs can serve as critical tools to reduce diagnostic errors, traumas, and inequities by providing a structured means for patients to communicate their expectations and experiences on diagnoses.^1^ By doing so, PRMs ensure that the diagnostic process reflects the reality of the patient’s condition, especially in diagnostic uncertainties, rather than relying solely on clinical judgment, which may be prone to bias or gaps, especially in diverse patient populations across different medical contexts.

 One of the key strengths of McDonald and colleagues’ argument is the focus on the variability in patients’ abilities to co-create diagnostic excellence.^1^ Patients are not a homogeneous group; their capacity to engage with healthcare providers and articulate their experiences varies significantly based on factors such as age, socioeconomic status, education, and health literacy. In care for older adults, for instance, patients may have cognitive impairments or sensory limitations that make it difficult to fully participate in the diagnostic process. Similarly, in maternity care, language barriers, cultural differences, and psychological traits may hinder a patient’s ability to communicate their symptoms or concerns effectively during diagnostic pathway. To address these variations, PRMs must be developed in ways that are accessible and tailored to the specific needs of different patient groups.

## Enhancing Diagnostic Excellence in Maternity Care

 McDonald et al emphasizes the importance of integrating PRMs to address uncertainty and improve diagnostic outcomes—a perspective that strongly aligns with the realities of maternity care.^1,3^ While communication barriers and emotional factors influence diagnostic processes across many medical contexts, maternity care represents a particularly sensitive setting where diagnostic excellence oriented PRMs are especially critical. Pregnancy involves a complex and emotionally charged diagnostic journey, particularly in sequential screening and testing, where timely and informed decisions must be made under uncertainty. The dual responsibility of safeguarding both maternal and fetal health heightens the psychological burden, while variations in health literacy and communication capacity can exacerbate diagnostic inequities. By leveraging equity-informed diagnostic excellence–oriented PRMs, maternal healthcare systems can create a more transparent and supportive diagnostic environment, improving the quality of communication between expectant mothers, families, and healthcare providers, supporting shared decision-making, improving diagnostic experience, thereby achieving diagnostic excellence. This directly resonates McDonald and colleagues’ call for diagnostic processes grounded in the patient’s perspective, especially in emotionally sensitive and potentially inequitable healthcare settings.

 Particularly, in prenatal diagnosis, also known as prenatal screening and testing (PreST),^4,5^ where various medical techniques and methods—each with its own advantages and limitations, such as the assessment of nuchal translucency by ultrasound scanning, blood test measuring certain substances (eg, alpha-fetoprotein, human chorionic gonadotropin, estriol, and inhibin-A), non-invasive prenatal testing or cell-free DNA testing, amniocentesis, and chorionic villus sampling, are involved, women and their families from diverse backgrounds must navigate complex choices amid high levels of uncertainty. Equitable, personalized, and appropriate measures are needed in this specific diagnostic pathway to establish good communication patterns and provide pregnant women with clearer information, improving their understanding of prenatal test results and fostering more autonomous decision-making in the involved sequential stages.^5,6^ As women and families come from diverse backgrounds and have varying abilities to understand diagnoses and navigate uncertainties, developing PRMs for diagnostic excellence is especially valuable in PreST, where interpreting sequential results and addressing uncertainties are frequent and significant experiences. PRMs that help pregnant women articulate their concerns, understand the implications of test results, and participate in shared decision-making can reduce anxiety and improve the overall diagnostic experience. Thus, McDonald and colleagues’ framework^1^ for incorporating PRMs into diagnostic excellence measurement is applicable in this contexts, which could help to reveal diagnostic uncertainties, design equitable decision aids, enhance informed decision-making and reduce decisional conflict, a concern cautiously highlighted in public maternity care.^6-8^

 In the following, we illustrate how the McDonald and colleagues’ suggested framework and roadmaps of achieving diagnostic excellence and improving equity in the diagnostic pathway via PRMs^1^ can be applied in the process of PreST. This case highlights the importance of developing tailored PRMs to achieve equity of diagnostic excellence, especially in uncertain situations and when improving communication is crucial.

###  Case: Navigating Uncertainty and Communication in PreST Via PRMs With a Lens of Equity

####  1. Context and Scenario

 A pregnant woman undergoes prenatal screening and receives a test result of first trimester screening indicating a slightly elevated risk for a chromosomal abnormality. This creates uncertainty and anxiety, and the woman struggles to understand the implications of the test results and has to make a decision among various options (opting in the further testing or sitting out; selecting testing approaches). The clinician, meanwhile, has difficulty gauging the patient’s emotional response and addressing her concerns adequately. The communication gap makes it challenging for the patient to make informed decisions about further testing or care.

####  Goals of Patient-Reported Diagnostic Excellence in PreST

 The goal of patient-reported diagnostic excellence in PreST is to capture pregnant women’s experiences, expectations, and satisfaction throughout the screening process, with particular attention to communication effectiveness and decision aids, especially when results are uncertain. This involves evaluating routine screenings to improve communication, reduce anxiety associated with diagnostic uncertainty, and enhance overall support. PRMs would assess the patient’s understanding of results, emotional state, decisional conflicts, and preferences for further testing or consultations, providing a comprehensive view of the patient journey to support better, more empathetic care.

####  Tailored Patient-Reported Diagnostic Excellence in PreST (Examples of Diagnostic Excellence-Based PRMs)

 PRMs for diagnostic excellence in PreST focus on capturing key areas of the patient experience, such as satisfaction with the pathway, clarity of communication, handling of uncertainties, as well as emotional support. Patients’ satisfaction with the pathway could be assessed through questions like, “How satisfied were you with the explanation of the screening tests you received?” Clarity and transparency in communication and uncertainty management are crucial, with questions exploring whether patients clearly understood each step, including timelines for results and follow-ups. Emotional support is essential, especially during the waiting period, where questions like “Were you provided with adequate emotional support while waiting for your screening results?” will be valuable. These PRMs include elements that mainly assess anxiety levels and decisional conflicts. The structure of these PRMs includes a Likert scale for quantitative responses and open-ended questions for deeper insights, combining both quantitative and qualitative measures to capture a broad spectrum of patient experiences.

####  Co-creating PRMs With Patients, Families, and Healthcare Providers

 It’s essential to engage stakeholders by co-creating effective PRMs with patients, families, and healthcare providers. Involving pregnant women and families who have undergone screening offers valuable insights into their experiences, concerns, and emotional responses. Including healthcare providers—such as obstetricians, midwives, and genetic counselors—ensures that the PRM aligns with clinical needs and accurately captures key diagnostic dimensions. Considering health systems and advocacy groups helps make PRMs accessible and equitable across diverse patient populations. This collaborative approach ensures that PRMs are relevant and comprehensive, addressing all facets of the prenatal experiences and incorporating patient and clinician perspectives to address real challenges in diagnostic communication and decision-making. An additional benefit of co-creating PRMs is the opportunity to assess and mitigate the practical burden of completing and using these measures, ensuring their implementation does not place undue strain on either patients or providers.

####  Implementing and Integrating PRMs for Enhanced Diagnostic Excellence in PreST

 To achieve diagnostic excellence in PreST, PRMs should be integrated into routine care by linking responses to electronic health records, enabling providers to access patient feedback alongside clinical data. Training is crucial for interpreting PRM results and applying them to clinical decision-making. These insights can inform adjustments to care practices—enhancing communication, emotional support, and shared decision-making, particularly in the face of diagnostic uncertainty. Regular evaluation of these improvements, along with ongoing feedback from patients and providers, will help refine PRMs and ensure their continued relevance in evolving prenatal care contexts.

####  Roadmap of Applying PRMs to Achieve Diagnostic Excellence in PreST

Figure presents a roadmap adapted from McDonald and colleagues’ proposed framework and tailored to achieving diagnostic excellence in PreST through the strategic application of PRMs. The roadmap focuses on two primary objectives: #ScreenEval, which addresses routine screening experiences and communication clarity; and #DxStories, which supports patients in articulating their diagnostic journeys. Structured over a 12-year implementation timeline, the roadmap outlines four key phases—Developing PRMs, Endorsing PRMs, Implementing and Scaling Up, and Acting Upon Measures—each represented by sequential steps marked with visual indicators of consistency across goals. While synergies (green triangles) represent opportunities to align with existing routines, systems and initiatives, challenges (purple triangles) indicate potential barriers that may impede the development and implementation of diagnostic excellence–oriented PRMs in PreST.

**Figure:**
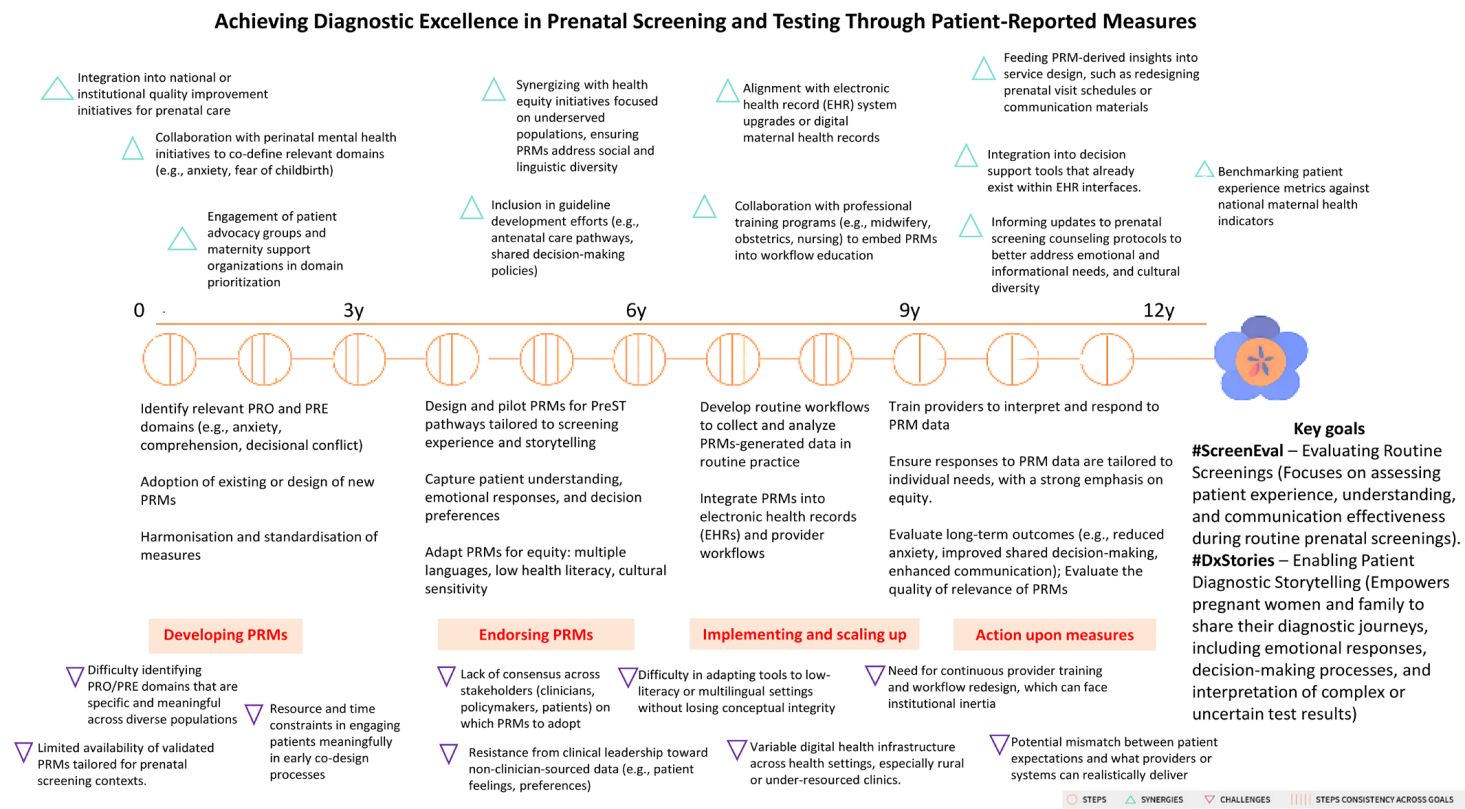
Achieving Diagnostic Excellence in Prenatal Screening and Testing Through Patient-Reported Measures. This figure visualizes the implementation of PRMs to achieve diagnostic excellence in PreST, structured around two key goals: 1) #ScreenEval and 2) #DxStories. The roadmap outlines four key phases over a 12-year implementation timeline. Each circle along the timeline represents a core step, with the number of vertical lines indicating the consistency of that step across both goals. Green triangles denote synergies—opportunities for alignment with existing systems or strategies—while purple triangles indicate key challenges that may hinder progress. Abbreviations: PRMs, Patient-Reported Measures; PreST, prenatal screening and testing; PRO, patient reported outcome; PRE, patient reported experience.

## Other Reflections

 Achieving diagnostic excellence through PRMs requires more than simply adopting the tool. It necessitates an equitable approach that accounts for the varying abilities and resources of patients to participate in the diagnostic process. Particularly, those from marginalized communities may face barriers such as language, cognitive impairments, or limited health literacy to respond to PRMs. Therefore, PRMs must be developed with equity in mind, ensuring that all patients, regardless of their background or abilities, can effectively make inputs, contribute to their diagnostic journey and co-work for diagnostic excellence.

 Maternal healthcare involves a broader spectrum of diagnostic activities beyond PreST, including the screening and monitoring of gestational diabetes, hypertensive disorders, anemia, and other perinatal complications. Applying PRMs to these domains could further advance diagnostic excellence by capturing patients’ experiences related to symptom recognition, interpretation of test results, and subsequent decision-making processes. Future research may extend this framework to address additional diagnostic touchpoints across the full continuum of maternal care.

## Conclusion

 By placing patients at the center of the diagnostic process and ensuring that their voices are heard through PRMs, McDonald et al offers valuable insights in achieving equity-focused diagnostic excellence. Their proposed roadmaps outline a clear strategy for leveraging PRMs in contexts where patients have varied abilities to participate in their care. By addressing the variability in patients’ capabilities to co-create diagnostic excellence and developing PRMs that are tailored to the specific needs of different populations, healthcare providers can ensure that all patients are able to actively participate in the diagnostic process. In doing so, PRMs not only improve diagnostic accuracy but also enhance patient satisfaction, reduce uncertainty, and facilitate better communication between patients and healthcare providers. In a broader view, McDonald and colleagues’ work serves as a call to action for healthcare systems to integrate patient-centered tools in ways that not only improve diagnostic precision but also ensure that diagnosis is accessible, useful, and inclusive for all, especially those traditionally overlooked by the healthcare system.

## Acknowledgements

 We thank the editors and reviewers for their valuable feedback and guidance during the development of this commentary.

## Ethical issues

 Not applicable.

## Conflicts of interest

 Authors declare that they have no conflicts of interest.

## Disclaimers

 This commentary reflects the authors’ perspectives on McDonald and colleagues’ work—“Achieving Diagnostic Excellence: Roadmaps to Develop and Use Patient-Reported Measures With an Equity Lens,” and it is not an exhaustive review of the topic.
